# Polo-like kinase inhibitor volasertib marginally enhances the efficacy of the novel Fc-engineered anti-CD33 antibody BI 836858 in acute myeloid leukemia

**DOI:** 10.18632/oncotarget.23880

**Published:** 2018-01-03

**Authors:** Bhavani Gopalakrishnan, Carolyn Cheney, Rajeswaran Mani, Xiaokui Mo, Donna Bucci, Alison Walker, Rebecca Klisovic, Bhavana Bhatnagar, Katherine Walsh, Bjoern Rueter, Irene C. Waizenegger, Karl-Heinz Heider, William Blum, Sumithira Vasu, Natarajan Muthusamy

**Affiliations:** ^1^ Comprehensive Cancer Center, The Ohio State University, Columbus, OH, USA; ^2^ Center for Biostatistics, Department of Biomedical Informatics, The Ohio State University, Columbus, OH, USA; ^3^ Division of Hematology, Department of Internal Medicine, The Ohio State University, Columbus, OH, USA; ^4^ Department of Hematology and Medical Oncology, Emory University, Atlanta, GA, USA; ^5^ Boehringer Ingelheim Pharma GmbH, Biberach/Riss, Germany; ^6^ Boehringer Ingelheim RCV, Vienna, Austria

**Keywords:** AML, BI 836858, volasertib, NK cells, CD33

## Abstract

Acute myeloid leukemia (AML) is the second most common type of leukemia in adults. Incidence of AML increases with age with a peak incidence at 67 years. Patients older than 60 years have an unfavorable prognosis due to resistance to conventional chemotherapy. Volasertib (BI 6727) is a cell-cycle regulator targeting polo-like kinase which has been evaluated in clinical trials in AML. We evaluated effects of volasertib in primary patient samples and NK cells. At equivalent doses, volasertib is cytotoxic to AML blasts but largely spares healthy NK cells. We then evaluated the effect of volasertib treatment in combination with BI 836858 on primary AML blast samples using antibody-dependent cellular cytotoxicity (ADCC) assays. Volasertib treatment of NK cells did not impair NK function as evidenced by comparable levels of BI 836858 mediated ADCC in both volasertib-treated and control-treated NK cells. In summary, volasertib is cytotoxic to AML blasts while sparing NK cell viability and function. Higher BI 836858 mediated ADCC was observed in patient samples pretreated with volasertib. These findings provide a strong rationale to test combination of BI 836858 and volasertib in AML.

## INTRODUCTION

Acute myeloid leukemia (AML) is the second most common type of leukemia in adults; it is a disease associated with aging with a peak incidence at 67 years [1]. In patients older than 60 years, it is associated with a particularly worse prognosis compared to younger patients, both due to intolerance and treatment resistance to chemotherapy [2–6]. Hence there is an urgent need for novel therapies for this group of AML patients. Recently, cell cycle regulators especially polo like kinase (PLK) family members have been an attractive therapeutic target in cancer biology [7–9]. Polo like kinase 1 (PLK1) is a mitotic serine, threonine kinase and plays a crucial role in G2/M transition and mitosis [9–11]. It is over expressed in neuroblastoma [12], and leukemias [8, 13, 14] with lower expression in untransformed cells. In our present study, we evaluated volasertib (BI 6727) a potent and selective small molecule inhibitor of PLK1. Volasertib is a low-molecular-weight, adenosine triphosphate–competitive kinase inhibitor that potently inhibits PLK1 as well as the two closely related kinases, PLK2 and PLK3, with 50% inhibitory concentration values of 0.87, 5, and 56 nmol/L, respectively [15–16]. Recently, volasertib was shown to have higher event-free survival in a randomized phase 2 trial where volasertib was given in combination with low-dose cytarabine in AML patients [18].

Another class of agents that have been studied in AML are antibodies targeting CD33, an antigen widely expressed on the surface of myeloid blasts. CD33, also called as Siglec-3 is a 67 kDa glycoprotein of sialoadhesin family and member of sialic acid is expressed in 90–95% of AML patient blasts [19–22]. Gemtuzumab Ozogamicin (GO) is an immunoconjugate consisting of a humanized anti-CD33 monoclonal antibody linked to calicheamicin, a potent antitumor antibiotic; it initially gained regulatory approval in 2000 but was voluntarily withdrawn from the market in 2010 due to safety concerns of tolerability when given in combination with conventional chemotherapy [23, 24]. However, randomized phase III trials have shown an improvement in overall survival resulting in renewed interest in CD33 targeted therapies [25]. This has prompted the development of CD33 antibodies without conjugation of cytotoxic drugs in hopes to minimize toxicity. Engineered Fc-domains with increased affinity for human Fcγ receptors have been shown to enhance potency and efficacy of effector cell mediated lysis [26]. More generally, clinical data have demonstrated the relevance of antibody-dependent cellular cytotoxicity (ADCC) for therapy of B-cell lymphoma with the CD20-specific monoclonal antibody rituximab [27]. Currently we are evaluating BI 836858 (mAb 33.1), a fully human IgG1 antibody specific for human CD33 which is Fc-engineered for increased binding to FcγRIIIa and which mediates ADCC. Our initial preclinical studies show that BI 836858 efficiently mediates ADCC on AML cell lines and primary AML cells. The specific cytolysis caused by BI 836858 is significantly higher than that of Lintuzumab (HuM195), a conventional, non Fc-engineered CD33-specific antibody which has been evaluated in clinical trials [28]. BI 836858 as an emerging immunotherapeutic option in AML led us to investigate its effect in combination with volasertib.

We have evaluated the effect of two different agents; a novel Fc-engineered anti-CD33 antibody (BI 836858) and volasertib (BI 6727) in primary AML blasts samples. Volasertib does not adversely impact NK cell viability and function. We observed greater BI 836858 mediated ADCC in volasertib-treated AML patient samples when compared with control-treated samples. Thus, our initial preclinical data show that volasertib treatment does not impair ADCC of the CD33 monoclonal antibody BI 836858 on primary AML cells. Hence a combination of BI 836858 and volasertib could result in additive or synergistic therapeutic effects.

## RESULTS

### Effect of volasertib on primary AML blasts

We initially investigated the effect of volasertib at different concentrations and time points on various AML cell lines (MV4-11, Kasumi-1, THP-1 and HL-60) in a MTS assay and the results obtained are comparable to recently published data [17] (data not shown). To attempt to mimic physiological concentrations achieved in patients, we selected doses of 50 nM, 500 nM and 1000 nM for all our studies described here.

**Figure 1:**
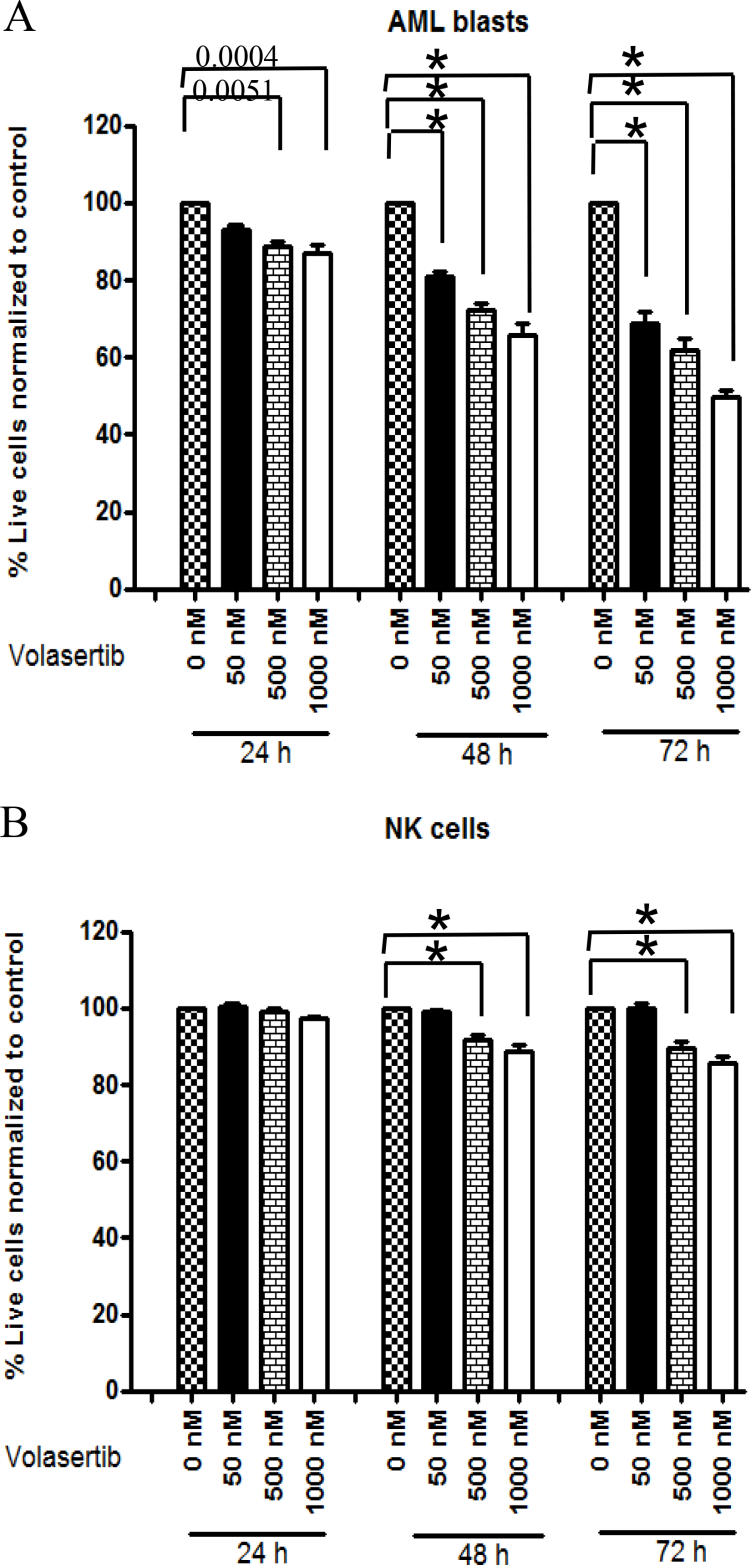
Volasertib is cytotoxic to AML blasts but at equivalent concentration spares or is less cytotoxic to NK cells AML blasts cells (2 × 10^6^) or NK cells (1 × 10^6^) were treated with the indicated concentrations of volasertib (50 nM, 500 nM, 1000 nM) for various durations (24 h, 48 h, 72 h) and viable cells were defined as the Annexin V-FITC negative and PI negative population. Viable cells in each sample were expressed as percentage of Annexin V negative/PI negative cells normalized to untreated control cells (0 nM volasertib). (**A**) AML blasts (*n* = 9) and (**B**) NK cells (*n* = 11). In all panels, ^*^ indicates *p* < 0.0001 compared to control as determined by mixed effect statistical model.

We analyzed viability of primary AML blasts after treatment with volasertib using dual staining with Annexin-V-FITC and Propidium iodide. The viable cells in each sample were expressed as % Annexin V negative/PI negative cells normalized to control-treated samples. Analysis of nine different primary AML samples revealed that volasertib, at concentrations from 50 nM to 1000 nM, exerted dose- and time-dependent direct cytotoxicity. For instance, a 30% decrease in viability at 50 nM (*p* < 0.0001, 95% CI-11.2, 18.7%) and 50% decrease in cell viability (*p* < 0.0001, 95% CI-18.9–26.5) at 1000 nM were observed after 72 hrs when compared to control (Figure 1A).

### Effect of volasertib on healthy NK cells

Treatment of NK cells from healthy donors (*n* = 11) with 50 nM of volasertib failed to mediate cytotoxic effects at any time point (*p* = 0.53) tested compared to controls (Figure 1B). However, higher doses of volasertib treatment after prolonged incubation of 72 hrs resulted in moderate decrease in viability (15% decrease, *p* < 0.0001, CI-7.9-11.5%) (Figure 1B). At 50 nM, volasertib was cytotoxic to AML blasts (70% viability) *vs* NK cells (100% viability); at higher concentrations, volasertib was also cytotoxic to NK cells, although toxicity was lower as compared to AML blasts (median 60% viability for AML blasts vs 90% viability for NK cells at 500 nM, 50% viability for AML blasts *vs* 90% viability for NK cells at 1000 nM (Figure 1A and 1B, *p* < 0.001). Thus, at equivalent concentrations, volasertib is more cytotoxic to AML blasts compared to NK cells.

### Volasertib treatment enhances BI 836858 mediated ADCC and is most pronounced in samples with higher expression of CD33

**Figure 2:**
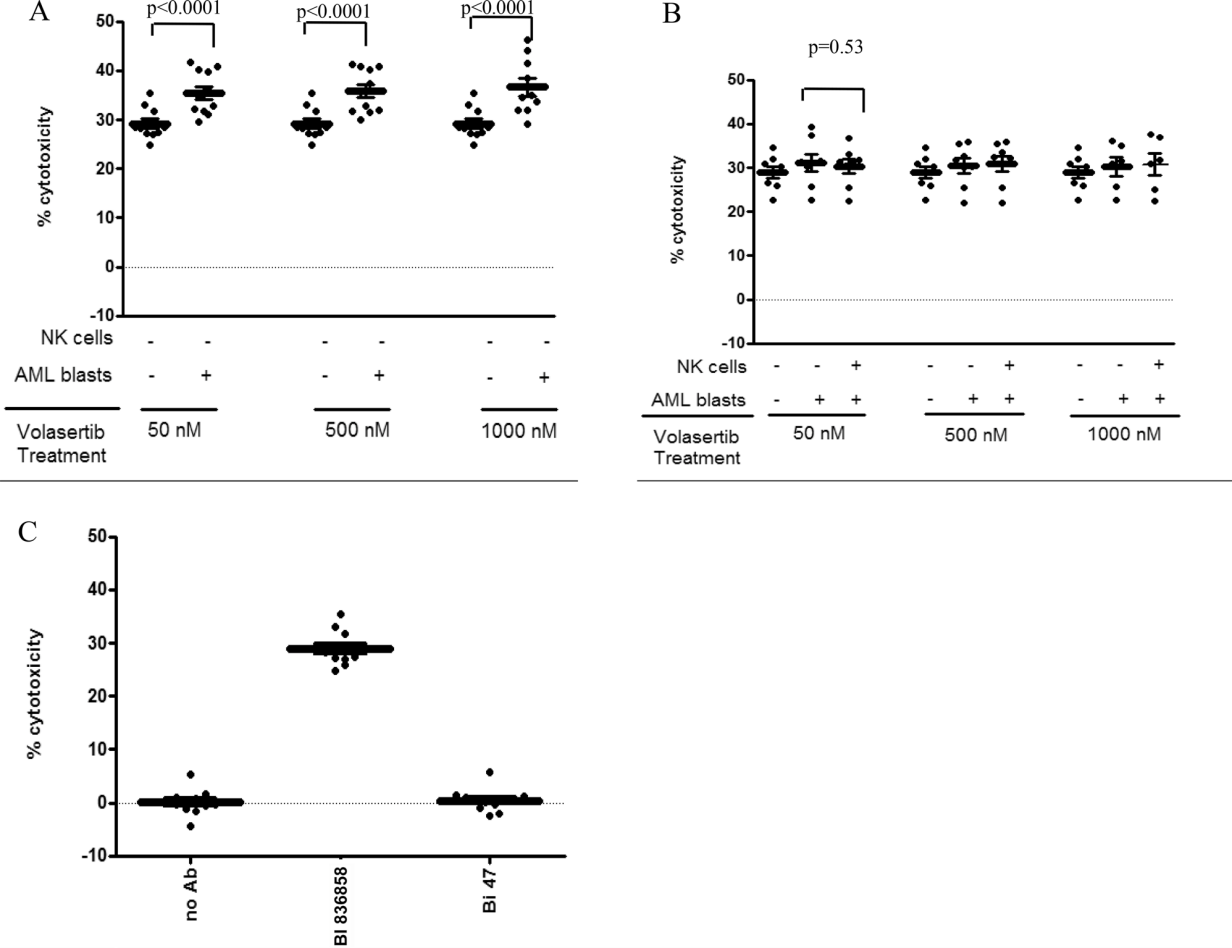
Volasertib treatment increases BI 836858 mediated ADCC and is most pronounced in samples with higher expression of CD33: Effector cells were allogeneic NK cells from healthy donors applied at an E: T ratio of 12:1, target cells were primary AML blasts Antibody concentration was 10 µg/mL. Panels (**A**–**C**) had primary AML blasts as targets and healthy donor NK cells as effectors. In panel A and B, the “-“and “+” symbols represent whether volasertib was added to AML blasts or NK cells. Panel A shows the relative cytotoxicity (%) for a subset of AML blasts that showed increased CD33 expression (*n* = 5). The effect of volasertib at various concentrations (50 nM, 500 nM, 1000 nM) on primary AML samples (left panel) and NK cells (right panel) is shown. Panel B shows the ADCC of BI 836858 for a subset of AML blasts that did not show increase in CD33 expression after volasertib treatment. The effect of volasertib at different concentrations (50 nM, 500 nM, 1000 nM) for pre and post volasertib treated AML blasts and NK cells are shown as relative cytotoxicity (%). In panel B, volasertib-treated NK cells did not show decline in ADCC compared to untreated NK cells. Panel C represents percentage of cytotoxicity of BI 836858 (CD33) as compared to BI 836847 (BI47, Fc-engineered isotope control antibody) and no antibody when ADCC was performed using untreated AML blasts and NK cells. ADCC experiments were performed in triplicate. In all panels, ^*^indicates *p* < 0.0001 compared to control as determined by mixed effect statistical model.

We investigated the effect of volasertib on the ADCC function of the CD33-directed monoclonal antibody BI 836858. AML blasts (*n* = 10) were treated with 50 nM of volasertib for 24 hrs and we identified five patients that showed upregulation of surface CD33 and five patients that showed minimal change in surface expression of CD33 following treatment. In samples with higher CD33 expression in response to volasertib, we observed higher BI 836858-mediated ADCC (Figure 2, Panel A). However no change in BI 836858 mediated ADCC was noted in samples where CD33 expression was unchanged or lower (Figure 2, Panel B). At an E:T ratio of 12:1, we observed increased cytotoxicity following pretreatment of AML blasts with 50 nM volasertib (36% *vs* 29%, *p* < 0.0001, 95% CI-5.1–8.4%) and 1000 nM volasertib (38% *vs* 28%, *p* < 0.0001, 95% CI-6.1-9.9%) compared to control treated samples (Figure 2A). We tested for CD33 expression in an additional 10 samples with FLT3ITD mutations and did not observe an upregulation in CD33 expression.

### Pre-treatment of NK effector cells with volasertib did not compromise BI 836858 mediated ADCC

NK effector cells play an important role in BI 836858 mediated ADCC. Allogenic NK cells show increased cytotoxicity compared to autologous NK cells [20, 22]. While volasertib treatment of NK cells did not compromise their viability we tested if pretreatment of NK cells with volasertib would compromise the NK activity in the ADCC assay against AML targets. Allogeneic NK cells treated with volasertib concentrations up to 1000 nM for 24 h exhibited BI 836858-induced ADCC against AML blasts similar to volasertib untreated NK controls. Figure 2B shows the cytotoxicity of volasertib-treated AML blasts and NK cells compared to control treated cells, suggesting that volasertib treatment enhances BI 836858 mediated ADCC without altering NK cell function. Figure 2B shows ADCC in patient samples where no increase in CD33 expression after volasertib treatment was observed. ADCC was measured after 24 hr of treatment at all concentrations and E: T ratios were similar to AML blasts not treated with volasertib (*p* = 0.53). Also, in control-treated AML target cells and NK effector cells from healthy donors, BI 836858 showed increased cytotoxicity over control antibody BI 836847 against primary AML blasts (Figure 2C). These results suggest that an additional effect of volasertib on BI 836858 ADCC was seen only in samples where CD33 expression was upregulated, but the ADCC was not lower in samples where CD33 expression was lower.

## DISCUSSION

In this paper, we confirm previous findings that volasertib is cytotoxic to AML cell lines and primary blasts [17]. We also show that at similar concentrations of volasertib, NK cells were relatively spared compared to AML blasts. Despite lower viability of NK cells at high concentrations of volasertib (1000 nM), we observed that ADCC of BI 836858 mediated by NK cells was not lower with volasertib-treated NK cells.

CD33 is a myeloid differentiation antigen which is predominantly expressed on the cell surface of leukocytes of the myeloid lineage and with high frequency on myeloid leukemia cells, including acute myeloid leukemia (AML), chronic myeloid leukemia (CML) and myelodysplastic syndrome (MDS) [31–35]. BI 836858 represents an unconjugated CD33 specific, fully human monoclonal antibody with enhanced ADCC activity for improved lysis of AML cells and is being explored therapeutically in AML [28].

Our studies reported here convincingly showed that allogenic NK cell viability or ADCC function was not compromised by volasertib treatment. Due to limited availability of NK cells from AML patients, we could not isolate autologous NK cells and hence ADCC assays with autologous NK cells could not be performed. The incidental finding of upregulation of surface expression of CD33 after volasertib treatment is noteworthy. It is possible that PLK1 and related kinases may play a negative regulatory role for CD33 expression. Alternatively it is also possible that this may reflect the potential role of volasertib in arresting the cells in G2/M phase. Nevertheless, the volasertib effect on only a subset of AML cells is intriguing and warrants detailed analysis. A larger cohort of primary samples would be necessary to evaluate whether there is a correlation between specific molecular subtypes of AML and upregulation of CD33 expression. While volasertib associated CD33 upregulation was associated with higher BI 836858-mediated ADCC, it is interesting to note that even in samples where CD33 expression was unchanged or lower, BI 836858-mediated ADCC was not decreased.

Limitations of this study include the availability of data regarding molecular mutations and cytogenetic risk group in only 50% of the samples. Several investigators have observed higher expression of CD33 in both *NPM1* and *FLT3ITD* molecular subgroups [36, 37]. Additionally, 87.8% of AML blasts in a cohort of 319 patients were shown to express CD33 [38] . Recent findings showing that effects of gemtuzumab ozogamicin correlated with higher surface expression levels of CD33 [39] suggest that interventions that may increase surface expression of CD33 would be attractive for CD33- targeted immunotherapeutic strategies in AML.

CD33 is also expressed in myelodysplastic syndrome and chronic myelomonocytic leukemia [40] An ongoing trial targeting CD33 expressing myeloid-derived suppressor cells using the same antibody described in this article is ongoing [41].

Here, we report that BI 836858 mediates target-specific ADCC against AML blast cells and that pre-treatment of effector cells with volasertib did not alter the cytotoxic effect of BI 836858. These findings provide a strong rationale for the use of BI 836858 for AML treatment in combination with volasertib. We also show that single-agent volasertib is cytotoxic to AML blasts but spares NK cells supporting potential use of volasertib as a maintenance agent in patients with healthy effector cells, as seen post-remission either after consolidative chemotherapy or after allogeneic hematopoietic stem cell transplantation. In conclusion, volasertib treatment does not impair ADCC of the CD33 monoclonal antibody BI 836858. These studies provide a basis for rational combination therapies of BI 836858 and volasertib in AML patients.

## MATERIALS AND METHODS

### AML samples

Primary AML patient samples were used for the preclinical studies. Blasts were obtained from apheresed blood samples when newly diagnosed patients presented with high leukocyte counts and were collected under an Ohio State University institutional review board-approved protocol and cryopreserved until use.

### Culture of AML blasts

The AML blasts (2 × 10^6^ cells/mL) were cultured in RPMI 1640 media (Invitrogen #21870-100) supplemented with 10% heat-inactivated fetal bovine serum (Sigma-Aldrich #F4135), two mM L-glutamine (Invitrogen #25030-081), 56 U/mL penicillin, and 56 µg/mL streptomycin (Invitrogen #15140-163) at 37°C, 5% CO_2_. Additionally, the AML blasts were supplemented with 10ng/mL each of IL-3 (R&D Systems # 203-IL-010), GM-CSF (R&D Systems # 215-GM-010) and SCF (R&D Systems # 255-SC-010).

### NK cell isolation

For allogeneic assays, human NK cells were enriched from the peripheral blood of healthy donors (American Red Cross) with the RosetteSep NK-cell Enrichment Cocktail (StemCell Technologies, #15065 BC, Canada) and Ficoll-Paque Plus (Amersham, #17-1440-03) centrifugation. Isolated cells (1 × 10^6^ cells/mL) were incubated in complete RPMI 1640 media at 37°C.

### Drug treatment

NK cells and primary AML blasts (2 × 10^6^ cells/mL) were incubated in presence or absence of volasertib (Boehringer Ingelheim, BI 6727) (stock 5 mM, in 0.1% DMSO+media) at concentrations of 50 nM, 500 nM or 1000 nM respectively for 24h, 48h and 72h. The control-treated cells were suspended in vehicle containing 0.1% DMSO in media.

### Antibody-dependent cell-mediated cytotoxicity (ADCC) assay

ADCC activity was determined by standard 4-hour ^51^Cr-release assay (Perkin Elmer #NEZ030001MC) [29]. Briefly, the target AML blasts were loaded with 0.1mCi ^51^Cr per 10^6^ cells for 30 min, excess washed off, then were incubated with 10 µg/mL of indicated antibodies BI 836858 (mAb33.1) or BI 836847 (BI47, a non-binding Fc-engineered isotype control antibody) at 37°C for 30 minutes. The unbound antibodies were washed off and the target cells were added at 5 × 10^3^ cells per well of 96-well plates. NK cells were added with two different effector to target ratios (E: T), 6:1 and 12:1. The supernatant was removed after four hours and counted on a Perkin Elmer Wizard gamma counter (Waltham, Massachusetts, USA). The percentage of specific cell lysis was determined by: % lysis = 100 x (ER-SR)/ (MR-SR), where ER, SR, and MR represent experimental, spontaneous, and maximum release, respectively.

### Flow cytometry

The pre and post volasertib treated cells were suspended in flow buffer (PBS plus 1% FBS) and incubated with indicated fluorochrome-labeled antibodies at 4°C for 30 min and washed according to manufacturer’s instructions. The antibodies include CD33-PE (BDpharmingen, #555450), CD34-BV421 (Biolegend #303610), CD45 APC (BDpharmingen, #555485). The samples were run on a Gallios flow cytometer (Beckman Coulter, Miami, FL, USA) with excitation at 488 nm and detection at 575/30 nm for PE channel, excitation at 405 nm and detection at 450/40 nm for BV421 channel and excitation at 633 nm and detection at 660/20 nm for APC channel and were analyzed using Kaluza 1.2 software (Beckman Coulter). The CD33 expression was defined on live positive CD45^+^/CD34^+^ blasts.

### Cell viability and apoptosis

The cell viability was assessed by flow cytometry following dual staining of Annexin-V-FITC and Propidium iodide (BD Bioscience, San Jose, CA, USA). The staining was done using methods previously published [30].The viable cells were identified as both Annexin V-FITC negative and PI negative staining using Beckman Coulter EPICS XL cytometer (Beckman Coulter, Miami, FL, USA). The viable cells in each sample were expressed as % by normalizing Annexin V negative/PI negative cells to control treated samples at indicated time points.

### Statistics

Since the same sample from each patient was treated with different conditions, data were analyzed by mixed effect models, incorporating repeated measures for each patient. Multiplicity was adjusted by Holm’s method to control the family wise error rate at 0.05. Data were analyzed by using SAS 9.4 software (SAS, Inc; Cary, NC).
